# An endpoint visualization loop-mediated isothermal amplification (LAMP) for detecting bubaline theileriosis

**DOI:** 10.1186/s43088-022-00251-x

**Published:** 2022-05-13

**Authors:** Sanjeev Kumar, Sanjhi Paliwal, Vikrant Sudan, Daya Shanker, Shanker Kumar Singh

**Affiliations:** 1 Department of Parasitology, U. P. Pandit Deen Dayal Upadhyaya Pashu Chikitsa Vigyan Vishwavidyalaya Evam Go Anusandhan Sansthan (DUVASU), Mathura, 281001 India; 2 College of Biotechnology, U. P. Pandit Deen Dayal Upadhyaya Pashu Chikitsa Vigyan Vishwavidyalaya Evam Go Anusandhan Sansthan (DUVASU), Mathura, 281001 India; 3 Department of Clinical Medicine, U. P. Pandit Deen Dayal Upadhyaya Pashu Chikitsa Vigyan Vishwavidyalaya Evam Go Anusandhan Sansthan (DUVASU), Mathura, 281001 India

**Keywords:** Bubaline theileriosis, ITS gene, LAMP

## Abstract

**Background:**

Tropical theileriosis is a significant disease affecting the health and production levels of buffaloes in India. It is caused by an apicomplexan—*Theileria annulata.* The timely and accurate detection of infection is vital for implementing a mass vaccination or control programme in a given area under outbreak. Most of the literature concerned with diagnosis of theileriosis revolves around cattle, and practically, there are very limited assays available for detecting bubaline theileriosis. Loop-mediated isothermal amplification (LAMP) assay certainly amplifies the targeted deoxyribosenucleic acid (DNA) with a comparatively higher efficacy, rapidity and sensitivity. Alongside, minimal use of sophisticated instruments in performing LAMP assay is certainly an add on. The present study describes the application of LAMP assay in diagnosing tropical theileriosis in buffaloes alongside, its comparison with polymerase chain reaction (PCR) and blood microscopical examination.

**Results:**

No cross-reaction was seen with DNA of other haemoprotozoan. LAMP was compared with blood microscopy and PCR. LAMP detected infection in 27 out of 100 buffaloes, while blood microscopy and PCR detected disease in 16 and 24 buffaloes, respectively.

**Conclusion:**

The sensitivity, specificity and kappa value prediction of LAMP were found to be much higher than the PCR and blood microscopy. The present communication reports the first use of LAMP in detecting theileriosis in buffaloes in the world.

## Background

Tropical theileriosis, caused by *T. annulata,* hinders the diary animals from attaining optimum heath and production levels [1, 2]. The disease is particularly severe in the semiarid belt of India covering border areas of Uttar Pradesh and Rajasthan [3, 4]. This high prevalence in these areas is attributed to the favourable hot and humid environmental conditions that are very much conducive for tick vector growth [5]. Water buffaloes are often affected by the disease leading to serious production losses [6]. But most of the times, it is difficult to diagnose the disease in buffaloes as the animals are often carriers (post-treatment) or the disease runs in a chronic phase. Blood microscopy suffers from limitations of sensitivity. Serology is an excellent option in chronic animals, but it fails to detect whether the animal is presently infected or the animal is recovered and still showing serological titres for the disease [7]. Under such circumstances, molecular tests detecting the deoxyribosenucleic acid (DNA) of causative agent are considered best in detection of patent infection [8, 9]. In contrast, these molecular detection tests require sophisticated machines as a basic prerequisite. Loop-mediated isothermal amplification (LAMP) assays substitute the use of sophisticated machines and can be performed under isothermal conditions using a simple water bath or a heat block. The present communication describes the application of LAMP assay in diagnosing tropical theileriosis in buffaloes alongside, its comparison with polymerase chain reaction (PCR) and blood microscopy.

## Methods

### Topography of studied area, sample collection and ethical compliance

The studied area comes under semi arid zone of northern India and comprised of Mathura, Uttar Pradesh and borders of Rajasthan. The area is located at 27.49° N latitude and 77.67° E longitude. The studied area is considered endemic for tropical theileriosis [3, 10] as the semiarid weather favours the propagation of tick vectors [5]. Unrestricted movement of animals infested with ticks, across the open borders, further adds to spread of the disease.

**Fig. 1:**
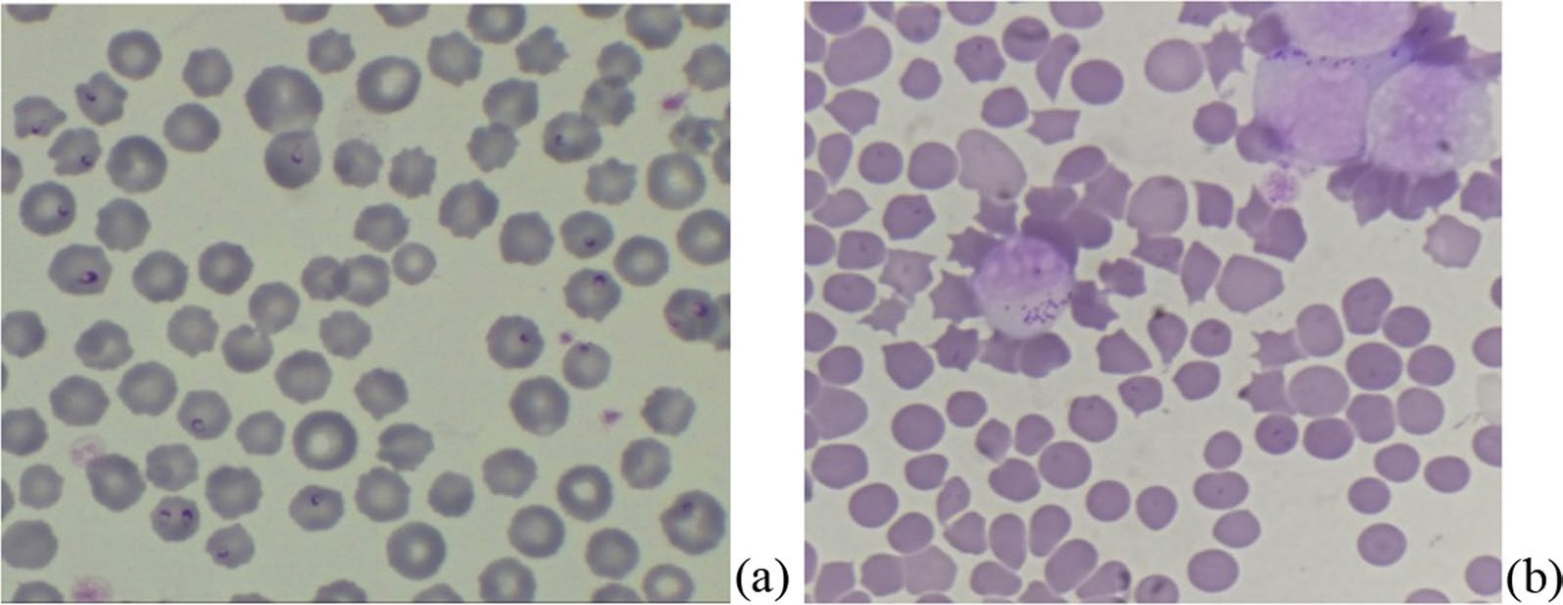
**a** Giemsa-stained blood smear showing *T. annulata* infected in RBCs (**a**) and lymphocyte (**b**) (100X)

Blood samples from 100 randomly selected buffaloes were collected in sterilized vacutainers. All the screened animals were adults (3–5 years of age) and apparently healthy without any history of any previous disease exposer in recent past (up to last 6 months). Blood was stored at − 20 °C till DNA was isolated. Alongside, thin blood smears were examined microscopically following Giemsa staining, for presence of intraerythrocytic piroplasm or Koch blue bodies (Fig. 1a, b). Collection of blood was done in accordance with the laid guidelines of Institutional Animal Ethical Committee, and the due permission was accorded via voucher number IAEC/17/23.

### DNA extraction, comparative LAMP assay vis-à-vis PCR

DNA was extracted using commercial DNA extraction kit (Promega^©^, USA) following the manufacturer’s protocol. Four primers (F3, B3, FIP, BIP) were custom synthesized from Imperial Life Sciences^©^, Gurugram, India [11]. The primers were firstly checked on Primer Explorer V4 program before ordering. The primer sequence consisted of F3: TGCACACAGTCATCTCAA; B3: GTGTGAGCCAAGACATCC; FIP: TTCACAAATCCAAATGGAAAGCTCTGAATTCGTCTACATTTTGTGGAATTGGT and BIP: ACAAGAGTTCAAGGACTAGAACCTGAATTCTAAATCCGAGTTACAAGGACC.

LAMP reaction was set up in a final volume of 25 µl and the reaction mixture comprised of 2.5 µl of 10X LAMP buffer (Imperial Life Sciences^©^), 1 µl of dNTP (0.4 mM), 0.5 µl of F3 and B3 primers (each 20 pmol), 2 µl of FIP and BIP primers (each 30 mol) 2 µl of MgSO4 (2 mM; Imperial Life Sciences^©^), 2 µl of betaine (0.4 M; Sigma Aldrich^©^) and 2 µl of DNA template. The volume was made 24 µl by adding nuclease-free water. This mixture was heated at 95 °C for 5 min followed by chilling on ice. Subsequently, 1 µl (8U) of *Bst* polymerase (Imperial Life Sciences^©^) was added to it, and the tube containing the reaction mixture was kept at 60 °C for 60 min. Finally, the reaction was terminated by heating the reaction mixture at 80 °C for 2 min. For comparing LAMP, PCR was also done on all the samples targeting *Theileria annulata* merozoite surface protein *(TAMS 1)* gene following the protocol of Paliwal et al.[12]. Initially, LAMP and PCR assays were laboratory standardized on known positive DNA of *T. annulata* (confirmed by sequencing, accession number: MH277611). Once standardized, the protocols were performed on individual blood sample collected from buffaloes. The positive amplification was seen at 785 bp specific for primers described previously. Genomic DNA from a known negative buffalo calf and nuclease-free water served as negative and no template controls, while the confirmed *T. annulata* DNA served as positive control.

### Visualization of LAMP and evaluation of LAMP vis-à-vis PCR and blood microscopy

The LAMP mixture tubes were removed after termination of reaction, and 1 µl of fluorescent intercalating SYBR green dye (Invitrogen^©^) was added for visualization of DNA accumulation in reaction tubes by visual fluorescence. The positive samples were visualized by change in colour of reaction mixture upon addition of dye. Further, the LAMP as well as PCR products were run on 1.5% agarose gel incorporated with ethidium bromide following electrophoresis.

The specificity of LAMP primers was checked using the known DNA of *Trypanosoma evansi, Babaesia bigemin, Theileria equi* and *Ehrlichia canis.* MedCalc software was used for calculating relative sensitivity and specificity of LAMP in comparison with PCR and blood microscopy. Finally, kappa values were calculated using GraphPad software.

## Results and discussion

### LAMP vis-à-vis PCR and blood microscopy

**Fig. 2:**
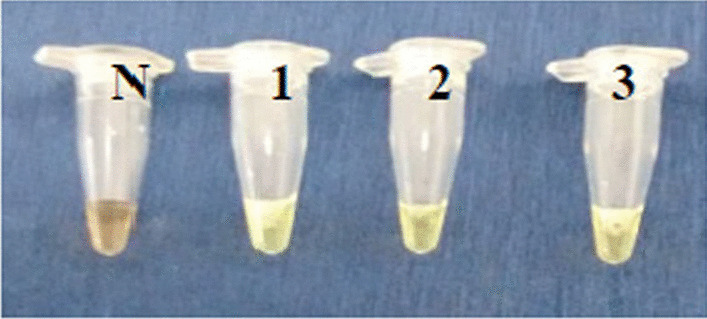
Visualization of LAMP products. Tube N: known negative sample. Tube 1: known positive sample. Tubes 2–3: test samples

**Fig. 3:**
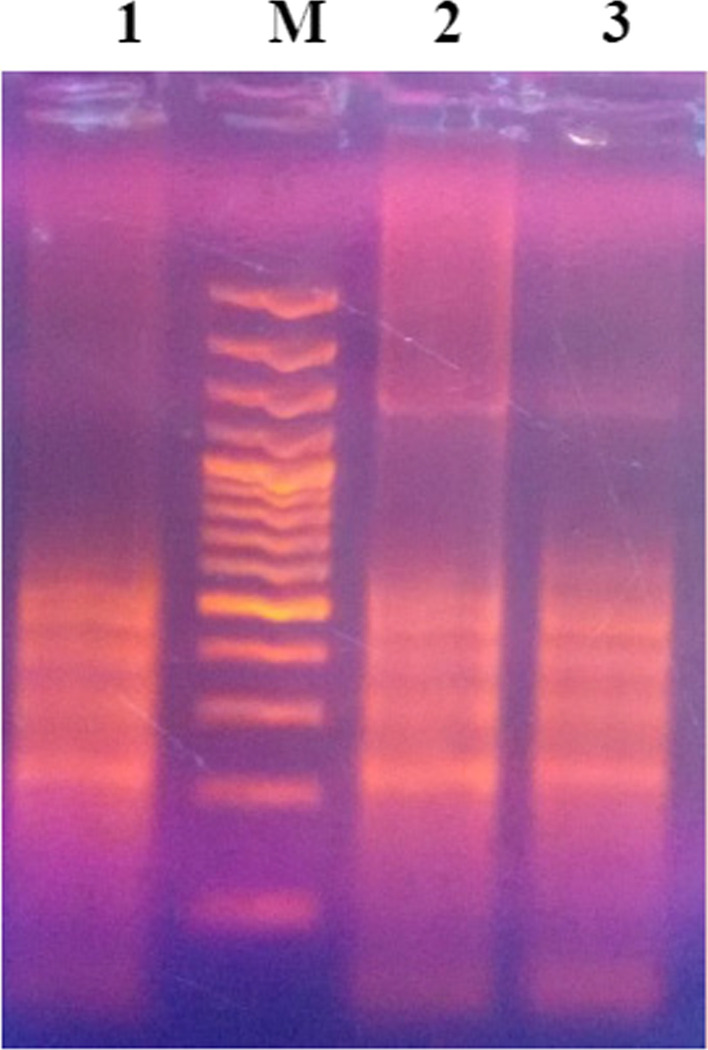
LAMP-based amplification of ITS gene. Lane M: 100-bp DNA ladder. Lane 1: positive *T. annulata* DNA. Lanes 2–3: test samples

Positive LAMP was analysed by visualization of DNA accumulation in reaction tubes by virtue of visual fluorescence (Fig. 2). The same was further confirmed by observing a specific ladder-like pattern upon electrophoresis (Fig. 3). No reaction was seen with DNA of other tested haemoprotozoa accounting for high specificity of LAMP primers. Alongside, negative and non-template controls did not give any visible florescence or ladder-like pattern.

**Fig. 4:**
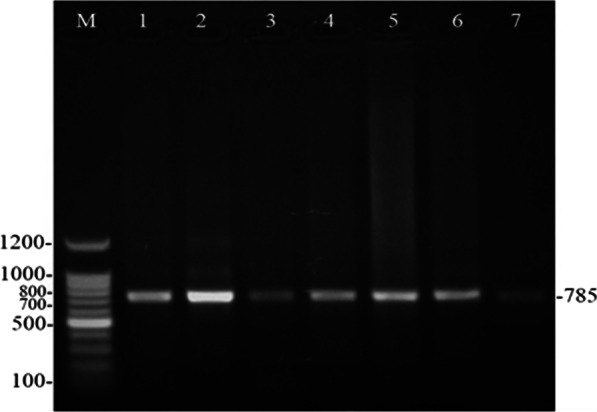
PCR amplification product of *TAMS* 1 gene. Lane M: 100-bp DNA ladder (Imperial Life Sciences, NEB, USA). Lanes 1–7: Field samples

Theileriosis was detected in 27 out of 100 buffaloes using LAMP, while PCR detected infection in 23 animals (Fig. 4). Blood smear was examination able to see piroplasm stage in 16 buffaloes. The relative efficacy of LAMP, PCR and blood smear examination in diagnosing tropical theileriosis is presented in Table 1. LAMP showed high sensitivity values of 94.81% (95% CI 87.23–98.57%) in comparison with PCR and a high kappa value of 0.894 (SE of kappa: 0.052; 95% CI 0.792–0.995).

**Table 1:** Statistical analysis of results of comparison of LAMP with PCR and blood smear examination using MedCalc's Diagnostic test evaluation calculator

TEST	PCR			Blood smear		
LAMP	Positive	Negative	Total	Positive	Negative	Total
Positive	23	4	27	16	11	27
Negative	0	73	73	0	73	73
Total	23	77	100	16	84	100

Statistics	Value	95% confidence interval	Value	95% confidence interval
Sensitivity	100.00%	85.18–100.00%	100.00%	79.41–100.00%
Specificity	94.81%	87.23–98.57%	86.90%	77.78–93.28%
Positive likelihood ratio	19.25	7.41–49.98	7.64	4.40–13.25
Negative likelihood ratio	0.00		0.00	
Disease prevalence	23.00%	215.17–32.49%	16.00%	9.43–24.68%
Positive predictive value	85.19%	68.89–93.72%	59.26%	45.61–71.62%
Negative predictive value	100.00%		100.00%	
Accuracy	96.00%	90.07–98.90%	89.00%	81.17–94.38%
Kappa	0.894		0.680	
S.E. of kappa	0.052		0.086	
95% confidence interval	0.792 to 0.995		0.511 to 0.849	

The environmental as well as rearing conditions of the India favour the survival and propagation of the vector and vector-borne pathogens in the livestock. Timely diagnosis and intervention is of paramount importance to minimize the economic losses in theileriosis. A suitable pen-side diagnostic assay further adds in minimizing the theileriosis-related production losses to the livestock sector. Further, rapid and accurate diagnosis is the basic prerequisite for any epidemiological study. Routine diagnosis of theileriosis is performed using blood microscopy and/ or lymph node biopsy [13]. Though blood microscopy is considered as gold-standard method, the method suffers drastically in carrier animals owing to less sensitivity [10]. Serological techniques such as indirect fluorescent antibody test (IFAT) [14], indirect enzyme-linked immunosorbent assay (ELISA) [15] and competitive enzyme-linked immunosorbent assay (cELISA) [16] suffer from the limitation in finding that if the infection is active or the serological titres are due to the persistence on antibodies post-recovery. Molecular probes are regularly used. But, again, they require sophisticated instruments. Nucleic acid-based assays, notably PCR [8, 10], and its types like nested [10] and duplex PCR [9], random amplified polymorphic DNA (RAPD) [10] and restriction fragment length polymorphism (RFLP) [17, 18], are widely used to identify for confirmation of theileriosis in animals. However, due to economic and /or practical reasons these methods may not always be available in low structural facilities. These limitations of PCR have inspired the development of platforms for the isothermal nucleic acid amplification technique. Among various assays, LAMP technique got the attention of the scientist all around the world for the diagnosis of diseases of animals and human. The assay is inherited with the high degree of simplicity, as it can be performed at a particular temperature and final amplified product can be detected visually in the tube [7]. A few reports of use of LAMP in diagnosing theileriosis in cattle are available [7, 11, 19]. Search of the literature revealed virtual absence of LAMP in detection of bubaline theileriosis. The present work is the pioneer step in this direction. In this current study, detection of *T*. *annulata* infection was done by amplifying the nucleic acid of the parasite in the variable temperature amplification assay, PCR and single-temperature amplification assay, LAMP. Carryover contamination is the main limitation of LAMP, which leads to false-positive results. To avoid contamination, in our LAMP reactions, all essential precautions were adopted, and proper sealing of tubes was done to avoid contamination. Overall positivity rates found in the instant study are considered to be comparatively higher, yet they can be very well justified owing to endemicity of theileriosis in the studied area [10]. Earlier work by Paliwal et al. [7] stated the efficiency of LAMP to be higher than simple PCR and almost at par with nested PCR. Herein also, the authors found higher efficiency of LAMP with regard to detecting capacity, sensitivity and specificity than the PCR assay.

## Conclusion

The present study was designed to evaluate a simple and convenient LAMP assay for field diagnosis of tropical theileriosis in buffaloes. ITS gene was targeted for this purpose. As the results suggested, LAMP was found to be sensitive than PCR and blood microscopy. Alongside, no cross-reaction with DNA of other haemoprotozoa supports the field level applicability of LAMP in diagnosing bubaline theileriosis.

## Data Availability

Data sharing is not applicable to this article as no datasets were generated or analysed during the current study.

## Abbreviations

LAMP: Loop-Mediated Isothermal Amplification
PCR: Polymerase Chain Reaction
DNA: Deoxyribosenucleic Acid
IFAT: Indirect Fluorescent Antibody Test
ELISA: Enzyme-Linked Immunosorbent Assay
cELISA: Competitive Enzyme-Linked Immunosorbent Assay
RAPD: Random Amplified Polymorphic DNA
RFLP: Restriction Fragment Length Polymorphism
TAMS: *Theileria annulata* Merozoite Surface protein
